# A comparison of the dental status and treatment needs of older adults with and without chronic mental illness in Sevilla, Spain

**DOI:** 10.4317/medoral.18332

**Published:** 2012-12-10

**Authors:** Eugenio Velasco-Ortega, Juan J. Segura-Egea, Sara Córdoba-Arenas, Alvaro Jiménez-Guerra, Loreto Monsalve-Guil, José López-López

**Affiliations:** 1 Department of Stomatology, (section of Gerodontology), School of Dentistry, University of Sevilla, Spain; 2 Department of Stomatology, School of Dentistry, University of Sevilla, Spain; 3 Department of Odontostomatology, School of Dentistry, University of Barcelona, Spain

## Abstract

Objectives: To study the dental status and treatment needs of institutionalized older adults with chronic mental illness compared to a non-psychiatric control sample. Study Design: The sample size was 100, in which 50 were psychogeriatric patients (study group; SG) classified according to DSM-IV, with a mean age of 69.6 ± 6.7 years, and 50 non-psychiatric patients (control group; CG), with a mean age of 68.3 ± 6.9 years. Clinical oral health examinations were conducted and caries were recorded clinically using the Decayed, Missing and Filled Teeth Index (DMFT). Results were analyzed statistically using the Student’s t-test or analysis of variance. Results: Caries prevalence was 58% and 62% in SG and CG, respectively. DMFT index was 28.3 ± 6.6 in SG and 21.4 ± 6.07 in CG (p < 0.01). Mean number of decayed teeth was higher in SG (3.1) compared to CG (1.8) (p=0.047). Mean number of missing teeth were 25.2 and 16.4 in SG and CG respectively (p<0.05). DMFT scores were higher in SG in all the age groups (p < 0.01). Mean number of teeth per person needing treatment was 3.4 in SG and 1.9 in CG (p= 0.037). The need for restorative dental care was significantly lower in the SG (0.8 teeth per person) than in the CG (1.7 teeth per person) (p = 0.043). Conclusions: Institutionalized psychiatric patients have significantly worse dental status and more dental treatment needs than non-psychiatric patients.

** Key words:**Gerodontology, oral health, older adult, psychiatric patients, schizophrenia.

## Introduction

The number of older adults is increasing worldwide and the global burden of oral disease among these older individuals is high. Despite a decline in edentulism among older adults in the past three decades, levels of dental caries remain high (1). The elderly population, in all countries and at all socioeconomic levels is also highly vulnerable to developing serious mental illness (SMI), including schizophrenia, depression and bipolar disorder. In Spain, like other Western countries, a significant number of persons with SMI require long-term hospitalization (2). A primary goal of a nation should be to improve the health and social functioning of its mentally ill population.

Hospitalized psychiatric patients are likely to constitute a high-risk group of individuals with respect to prevalence of dental disease and may require special attention. Suboptimal oral health has been self-reported among patients with SMI (3). Several reports detailing the oral health status of psychiatric patients in Denmark (4), The Netherlands (5), Italy (6), Spain (7), India (8), South Wales (9), Israel (10), United Kingdom (11) and Turkey (12) have revealed that mental illness and the medications used to treat the disorder can cause and magnify the severity of dental diseases with particular emphasis on caries experience (13).

The link between mental illness and dental caries has been attributed to the individual’s impaired ability to function which results in neglect of self-care, and in inadequate plaque control. In addition, the majority of psychotropic medications (e.g., phenothiazines, tricyclic antidepressants, and many others) frequently used to treat these disorders cause salivary gland hypofunction (14) producing xerostomia, which can lead to increased risk of dental caries (3). In addition to the xerostomic effect of medications used to treat individuals with depression, the illness itself is related to increased anticholinergic activity resulting in a magnification of hyposalivation and dry mouth (15). Elderly psychiatrically impaired individuals often have comorbid medical illnesses requiring the administration of additional xerostomia producing medications (16,17).

Patients receiving psychiatric medications that cause decrease salivary flow have a reduction in buffering capacity and dental caries occurs even with a normal diet. When these individuals perceive that their mouth is dry they often seek relief by sucking or chewing on candies which contain sugars, and thereby increase the risk of developing dental caries (18). Worsening the prevalence and severity of the caries is the fact that these individuals are often unable or unwilling to cooperate with dental treatment (19). Furthermore, the hospital nursing staff often lacks the necessary knowledge, abilities, and motivation to provide oral hygiene care to these patients (20).Little is known about the caries status of institutionalized Spanish psychiatric patients. An earlier study (21) investigated the dental health of an institutionalized psychiatric population in Spain, with a mean age of 58.0, reporting a mean DMFT index of 24.9. Another study (22) analyzed the prevalence of dental caries in an adult (age ranging from 20 to 40 years) Spanish population with mental disabilities, finding a mean DMFT index for the whole sample of 6.0. However, both studies lack a community based control group. Therefore, a descriptive cross-sectional study was undertaken to describe the caries experience of a group of institutionalized elderly psychiatric patients and to compare it to a group of individuals living in the community and free of psychiatric illness.

## Material and Methods

This study conformed to the ethical guidelines promulgated by the Department of Stomatology, Dental School, Sevilla University, Spain. The study group (SG) consisted of 50 subjects, aged 69.6 ± 6.7 years, 25 males and 25 females, who had been admitted to the Miraflores Psychiatric Hospital (Sevilla, Spain) ( Table 1). This long-term psychiatric hospital serves Sevilla County in Andalusia, southern Spain. The control group (CG) consisted of 50 individuals, aged 68.3 ± 6.9 years (p > 0.05), 24 males and 26 females, who consecutively presented as new patients seeking routine dental care (not emergency care) at the Gerodontology Unit, School of Dentistry , University of Seville. The criteria for inclusion in the control sample were that the patients were being seen for the first time at the school, were older than age 60 and that they were not taking psychotropic medications.

**Table 1:**
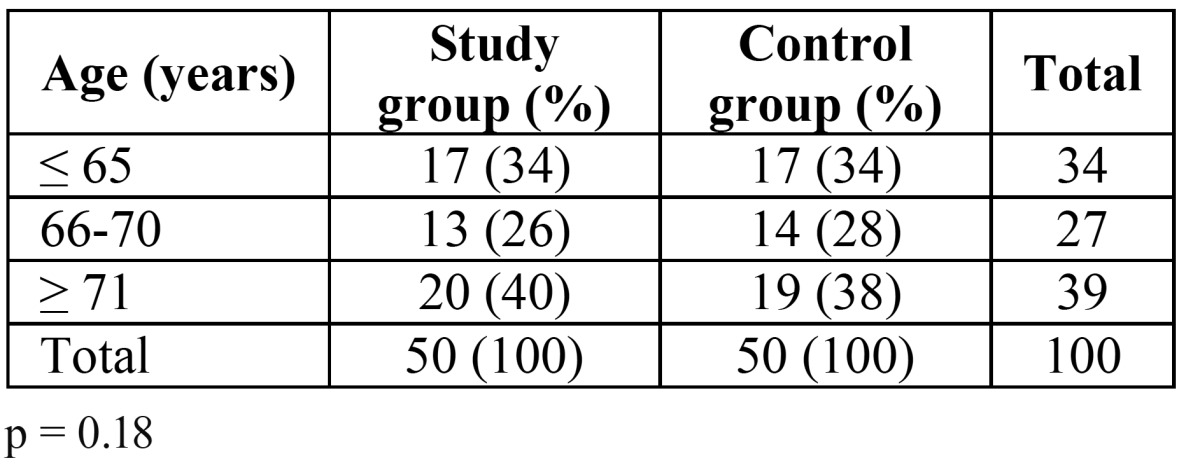
Distribution of patients in the study and control groups according to age.

-Data collection

Demographic data for the SG patients was obtained through interviews with the medical and psychiatric staff and similar information was garnered directly from members of the CG. The demographic variables included age, gender, and length of hospitalization (years). Psychiatric data from members of the SG was obtained by interviews with the psychiatric staff and a review of the patients' medical charts. Patients were classified according to the American Psychiatric Association Diagnostic and Statistical Manual of Mental Disorders (DMS), IV-TR (23) as suffering: 1) schizophrenia, 2) depression, or 3) other mental illness. The psychotropic medications were classified as phenothiazines, tricyclic antidepressants, benzodiazepines, or anti-Parkinsonian medications.

-Measures of dental caries

Caries status was determined according to crite¬ria set by the World Health Organization (24). Dental caries (either crown or root) was recorded clinically and documented by the Decayed, Missing and Filled Teeth (DMFT) Index. The decayed (D) tooth component identifies teeth with active dental caries; the missing (M) tooth component includes all teeth missing because of caries or other causes; and the filled (F) tooth component indicates teeth filled or restored because of dental caries. The DMFT Index measures the lifetime accumulation of dental caries experience, and the basis for the DMFT calculations were 32 teeth, i.e., all teeth including third molars (25).

Examinations were conducted by a team consisting of one dentist (S. C.) and two trained dental assistants, using mobile equipment.. A standardized dental explorer was used. Teeth were dried using air syringe, saliva ejector, and cotton rolls. Radiographs were not obtained because of ethical and practical reasons. Prior to the survey, the examining dentist was calibrated by an experienced clinical examiner (E.V.O.) and the inter-examiner kappa score was 81 percent.

-Statistical analysis

Raw data were entered into an Excel® spreadsheet (Microsoft Corporation, Redmond, WA, USA). Means and standard deviations were calculated for the number of teeth affected by caries, and mean values were evaluated statistically by the Student’s t-test or analysis of variance. Data were processed and analyzed using the SPSS 14.0 (SPSS, Inc., Chicago, IL, USA). Statistical significance was established at the p < 0.05 level.

## Results

-Psychiatric and medical data

In the SG, most of the patients suffered from schizophrenia (n = 28, 56%), one patient (2%) had depression, and the remainder (n = 21, 42%) had a variety of other mental illnesses. Psychotropic medications were taken by 76% of the subjects in the SG: 28 were taking benzodiazepines (56%), 27 phenothiazines (54%), 16 anti-Parkinsonian medications (32%), and 1 was taking antidepressants (2%). The mean number of psychotropic medications per patient was 1.9 ( Table 2). The mean length of hospitalization was 36 years (range: 6 - 62 years) ( Table 2). In the CG, none of the patients were taking psychotropic medications.

**Table 2:**
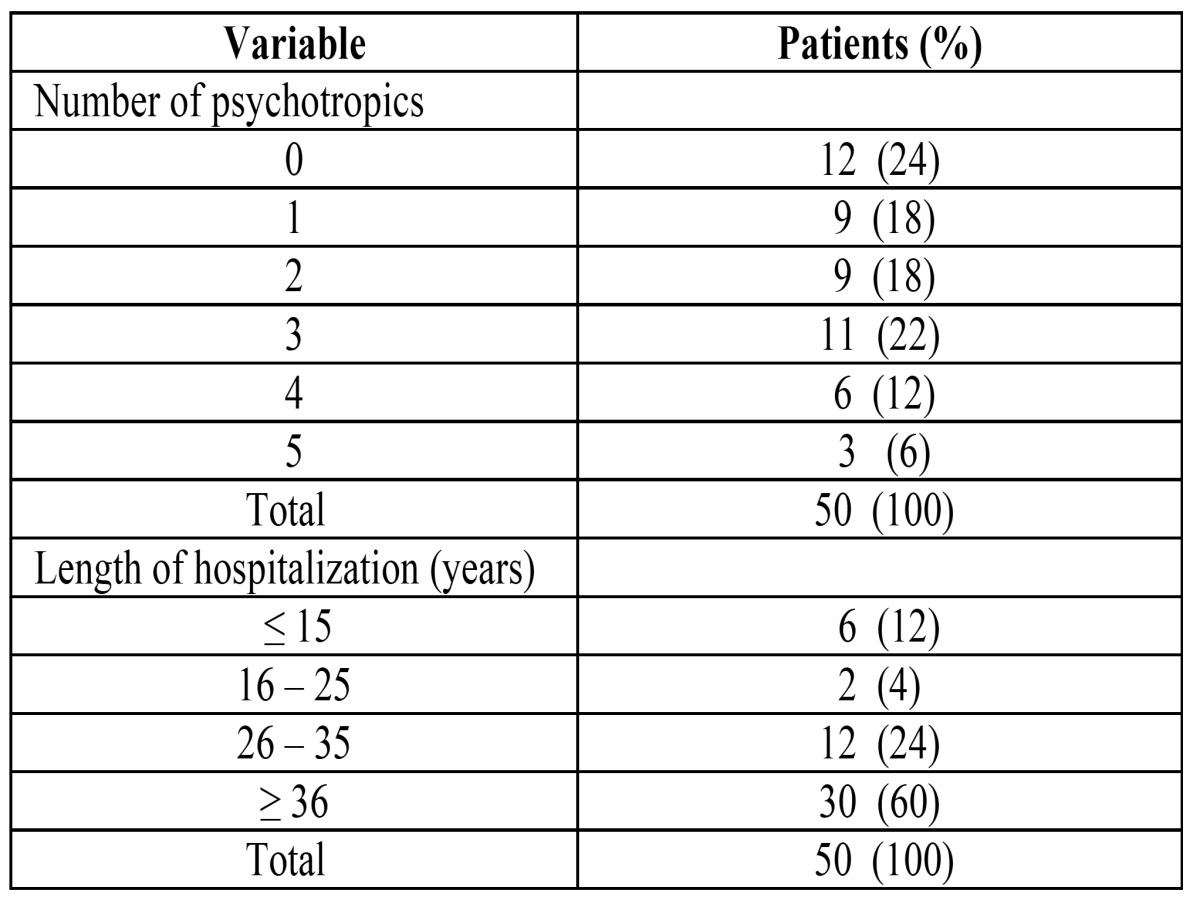
Patients distribution in the study group according to the intake of psychotropic medicaments and to the length of hospitalization.

-Dental caries experience

Caries was present in 29 patients (58%) in the SG and 31 individuals (62%) in the CG ( Table 3). However, among the individuals deemed caries-free because they were completely edentulous, 17 (34%) were in the SG and 4 (8%) were in the CG, and this difference was statistically significant (p<0.01).

**Table 3:**
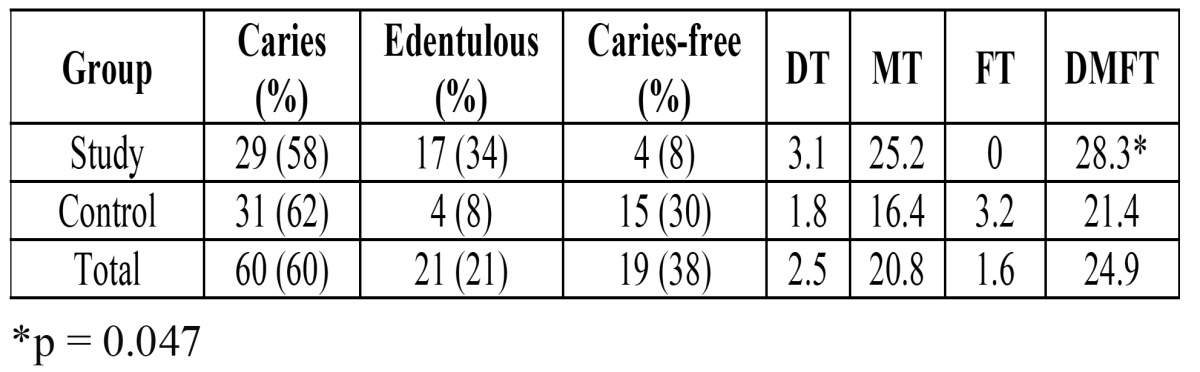
Caries experience in the study and control groups. DMFT Index with his components.

Significant differences were also detected in the values of the DMFT index between the two groups (SG = 28.3 ± 6.6 and CG = 21.4 ± 6.1; p < 0.01) ( Table 3). The mean number of decayed teeth was higher in the SG (DT = 3.1; range: 0 - 13.3) than in the CG (DT = 1.8; range: 0 - 13), and this was statistical significant (p = 0.047). In the SG 44 % of the patients manifested 3 or more decayed teeth, but in the CG only 28 % of the patients evidenced 3 or more decayed teeth. The mean number of missing (M) teeth was 25.2 and 16.4 in the study and control groups, respectively (p<0.05). None of the patients in the SG had filled teeth; in the CG the mean number of filled (F) teeth was 3.2.

Males had significantly higher decayed (D) teeth scores than females in both groups, but gender did not affect missing (M) teeth and filled (F) teeth scores. The D, M and DMFT scores were higher in the SG than in the CG in all the age groups (p < 0.01). Stepwise linear regression analysis showed that the mean DMFT index increased with age in the SG (p < 0.05), but not in the CG. The number of missing (M) teeth scores increased with the patients’ age (p<0.05) and length of hospitalization (data not shown).

-Treatment needs

The total treatment needs calculated were higher in the SG in both sexes and in all the age groups ( Table 4). The mean number of teeth per person needing treatment was 3.4 in the SG and 1.9 in the CG (p= 0.037). The need for restorative dental care was lower in the SG (mean need for patient = 0.8 teeth) than in the CG (mean need for patient = 1.7 teeth) (p = 0.043), because members of the SG had a higher mean number (25.2) of missing (M) teeth than members of the CG (16.4). The mean number of required extractions was 2.6 in the SG and 0.3 in the CG.

**Table 4:**
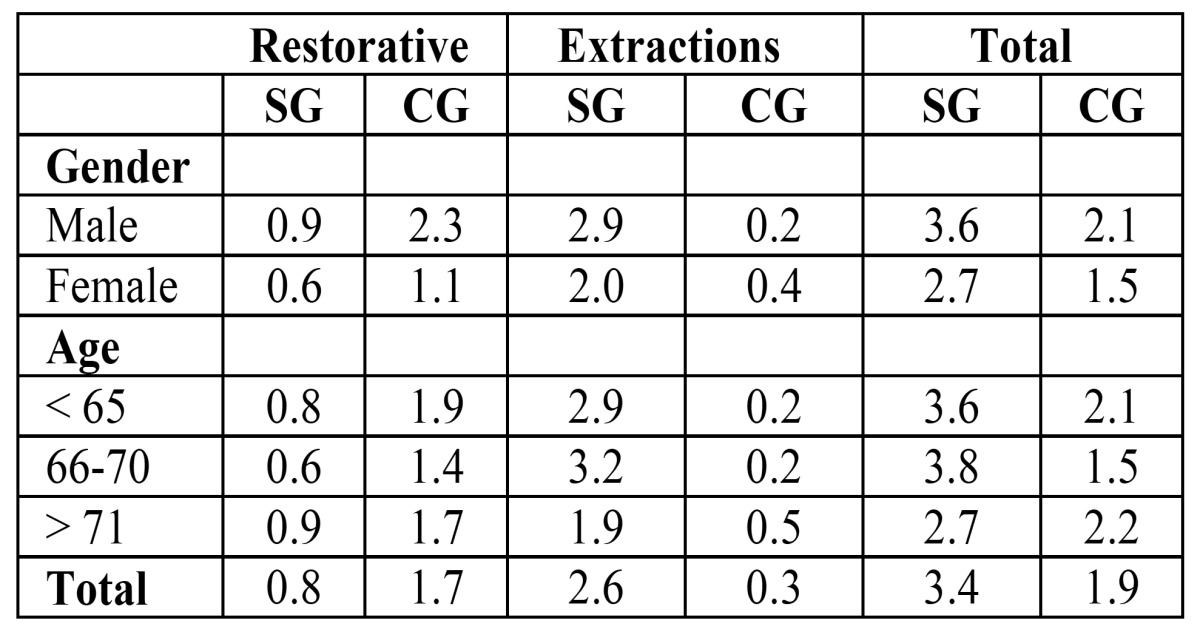
Treatment needs in both groups.

## Discussion

This study determined the caries experience and dental treatment needs of institutionalized Spanish elderly psychiatric patients and compared them to a gender and age-matched control group of community dwelling individuals attending a dental school in Seville. The methods of selecting subjects of the study and control groups were similar to other studies published previously (26,27).

The results of this study reveal a high prevalence of dental caries in both groups of patients. Approximately 60% of the total patients in our study had dental caries, but there remained significant differences between the DMFT values of the institutionalized elderly psychiatric patients (SG = 28.3 ± 6.6) (p < 0.01) when compared to the non-psychiatric impaired control population (CG = 21.4 ± 6.1). These findings are consistent with those of other studies which have noted an increased prevalence of dental caries among patients with chronic mental illness (4,10,13,20,28). Recently, the oral health of a group of Spanish schizophrenic outpatients and a control group without psychiatric illness have been evaluated, reporting higher scores to decayed, missing teeth, DMF-T index, and CPITN in the schizophrenic patients compared to the control group (29). The component of the DMFT index that exhibited the greatest difference between the two groups was missing (M) teeth. The mean number of missing teeth was significantly higher in the SG than it was in the CG (25.2 and 16.4, respectively) (p<0.05). This result can be ascribed to lack of hygienic dental habits between the two geriatric populations, as well as to the extracting of teeth rather that providing restorative dental treatment in psychiatric institutions, but rather extracting teeth (4,7,27,29). The results of our study are consistent with the work of Eustaquio et al. (26) who reported a DMFT index of 16.4 in the group of 65-74 year-old subjects. Of interest and at some variance with our results is the work of Chalmers et al. (27) who compared the caries experience for existing and new nursing home residents in Adelaide, Australia, and did not find significant differences between the groups as to their dentate status, tooth status (DMFT = 24.0 and 24.9, respectively), coronal caries experience, or root caries experience, with the exceptions that new residents had a significantly greater mean number of teeth, more filled coronal and root surfaces, and also new residents had significantly fewer decayed retained roots.

The present study shows a major number of teeth (5.9) with active caries without treatment, and, especially, the absence of filled teeth. On the contrary, Vigild et al. (30) reported an average of filled teeth of 4.8 in a sample of institutionalized elderly psychiatric patients in Denmark.

The stepwise linear regression analysis showed that the mean DMFT index increased with age in the study group (p < 0.05), but not in the control one. Previous reports have reported an age-dependent increase of DMFT index (30). The increase of the MT component with the age was significant in both groups. On the other hand, DT and FT components of the index tended to dimin-ish. Similar results were found by Zusman et al. (10) and Vigild et al. (30) who reported that the number of remaining teeth diminished with age, whereas the number of retained roots increased. Accordingly, most of the caries were located in these remaining roots. The progressive loss of teeth with age observed in the present study suggests that the Spanish elderly population suffers from inadequate treatment of dental diseases. Among dentate patients, we found cognitive impairment to be associated with a reduced number of teeth and an increased need for dental treatment. Other authors have found similar results (11).

In institutionalized psychiatric patients the carious disease process appears to progress over time and symptomatic teeth are extracted rather than restored, probably because of a lack of personnel available to provide preventive and restorative dental care. These findings are consistent with those seen in a similar population in Italy (6).

An analysis of our findings suggests that the extent of caries and need for dental treatment among institutionalized psychiatric patients is significantly greater than that of gender and age-matched non-psychiatric patients. There is a need to develop integrated dental and mental health care with emphasis on prevention of dental problems among older people.

## References

[1] Zeng X, Sheiham A, Bernabé E, Tsakos G (2010). Relationship between dental status and oral impacts on daily performances in older southern Chinese people. J Public Health Dent.

[2] Priebe S, Badesconyi A, Fioritti A, Hansson L, Kilian R, Torres-Gonzales F (2005). Reinstitutionalisation in mental health care: comparison of data on service provision from six European countries. BMJ.

[3] Kilbourne AM, Horvitz-Lennon M, Post EP, McCarthy JF, Cruz M, Welsh D (2007). Oral health in Veterans Affairs patients diagnosed with serious mental illness. J Public Health Dent.

[4] Hede B, Petersen PE (1992). Self-assessment of dental health among Danish noninstitutionalized psychiatric patients. Spec Care Dent.

[5] Ter Horst G (1992). Dental care in psychiatric hospitals in The Netherlands. Spec Care Dentist.

[6] Angelillo IF, Nobile CG, Pavia M, De Fazio P, Puca M, Amati A (1995). Dental health and treatment needs in institutionalized psychiatric patients in Italy. Community Dent Oral Epidemiol.

[7] Velasco E, Bullón P (1999). Periodontal status and treatment needs among Spanish hospitalized psychiatric patients. Spec Care Dentist.

[8] Kenkre AM, Spadigam AE (2000). Oral health and treatment needs in institutionalized psychiatric patients in India. Indian J Dent Res.

[9] Lewis S, Jagger RG, Treasure E (2001). The oral health of psychiatric in-patients in South Wales. Spec Care Dentist.

[10] Zusman SP, Ponizovsky AM, Dekel D, Masarwa AE, Ramon T, Natapov L (2010). An assessment of the dental health of chronic institutionalized patients with psychiatric disease in Israel. Spec Care Dentist.

[11] Purandare N, Woods E, Butler S, Morris J, Vernon M, McCord JF (2010). Dental health of community-living older people attending secondary healthcare: a cross-sectional comparison between those with and without diagnosed mental illness. Int Psychogeriatric.

[12] Gurbuz O, Alatas G, Kurt E, Issever H, Dogan F (2010). Oral health and treatment needs of institutionalized chronic psychiatric patients in Istanbul, Turkey. Community Dent Health.

[13] Stiefel DJ, Truelove EL, Menard TW, Anderson VK, Doyle PE, Mandel LS (1990). A comparison of the oral health of persons with and without chronic mental illness in community settings. Spec Care Dentist.

[14] Simmons DD, Al-Hashimi I, Haghighat N (2000). Effect of xerostomic medications on stimulated salivary flow rate in patients with Sjögren's syndrome. Quintessence Int.

[15] Friedlander AH, Marder SR (2002). The psychopathology, medical management and dental implications of schizophrenia. J Am Dent Assoc.

[16] Davies BM, Gurlan JB (1961). Salivary secretion in depressive illness. J Psychosom Res.

[17] Barnes GP, Allen EH, Parker WA, Lyon TC, Armentrout W, Cole JS (1988). Dental treatment needs among hospitalized adult mental patients. Spec Care Dentist.

[18] Gilbert GH, Heft MW, Duncan RP (1993). Mouth dryness as reported by older Floridians. Community Dent Oral Epidemiol.

[19] Yaltirik M, Kocaelli H, Yargic I (2004). Schizophrenia and dental management: review of the literature. Quintessence Int.

[20] Thomas A, Lavrentzou E, Karouzos C, Kontis C (1996). Factors which influence the oral condition of chronic schizophrenia patients. Spec Care Dentist.

[21] Velasco E, Machuca G, Martínez-Sahuquillo A, Ríos V, Lacalle J, Bullón P (1997). Dental health among institutionalized psychiatric patients in Spain. Spec Care Dentist.

[22] Rodríguez Vázquez C, Garcillan R, Rioboo R, Bratos E (2002). Prevalence of dental caries in an adult population with mental disabilities in Spain. Spec Care Dentist.

[23] Kawa S, Giordano J (2012 ). A brief historicity of the Diagnostic and Statistical Manual of Mental Disorders: issues and implications for the future of psychiatric canon and practice. Philos Ethics Humanit Med.

[24] Ishii T, Yoshida S (1978). Oral health surveys--basic methods--fundamental and practical problems of oral health surveys by WHO. Shikai Tenbo.

[25] Hunt RJ, Beck JD (1985). Methodological considerations in a dental epidemiologic survey of an elderly population. J Public Health Dent.

[26] Eustaquio MV, Montiel JM, Almerich JM (2010). Oral health survey of the adult population of the Valencia region (Spain). Med Oral Patol Oral Cir Bucal.

[27] Chalmers JM, Carter KD, Fuss JM, Spencer AJ, Hodge CP (2002). Caries experience in existing and new nursing home residents in Adelaide, Australia. Gerodontology.

[28] Orellana LM, Silvestre FJ, Martínez-Sanchis S, Martínez-Mihi V, Bautista D (2012 ). Oral manifestations in a group of adults with autism spectrum disorder. Med Oral Patol Oral Cir Bucal.

[29] Arnaiz A, Zumárraga M, Díez-Altuna I, Uriarte JJ, Moro J, Pérez-Ansorena MA (2011). Oral health and the symptoms of schizophrenia. Psychiatry Res.

[30] Vigild M, Brinck JJ, Christensen J (1993). Oral health and treatment needs among patients in psychiatric institutions for the elderly. Community Dent Oral Epidemiol.

